# Improved Cell Morphology and Surface Roughness in High-Temperature Foam Injection Molding Using a Long-Chain Branched Polypropylene

**DOI:** 10.3390/polym13152404

**Published:** 2021-07-22

**Authors:** Steven Mendoza-Cedeno, Mu Sung Kweon, Sarah Newby, Maksim Shivokhin, George Pehlert, Patrick C. Lee

**Affiliations:** 1 Department of Mechanical and Industrial Engineering, University of Toronto, 5 King’s College Road, Toronto, ON M5S 3G8, Canada; smendoza@mie.utoronto.ca (S.M.-C.); mkweon@mie.utoronto.ca (M.S.K.); 2 ExxonMobil Chemical Company, 5200 Bayway Drive, Baytown, TX 77520, USA; sarah.newby@exxonmobil.com (S.N.); maksim.e.shivokhin@exxonmobil.com (M.S.); george.j.pehlert@exxonmobil.com (G.P.)

**Keywords:** foam injection molding, chemical blowing agents, linear and branched polypropylene, surface roughness, foam structures, flexural properties

## Abstract

Long-chain branched polypropylene (LCB PP) has been used extensively to improve cell morphologies in foaming applications. However, most research focuses on low melt flow rate (MFR) resins, whereas foam production methods such as mold-opening foam injection molding (MO-FIM) require high-MFR resins to improve processability. A systematic study was conducted comparing a conventional linear PP, a broad molecular weight distribution (BMWD) linear PP, and a newly developed BMWD LCB PP for use in MO-FIM. The effects of foaming temperature and molecular architecture on cell morphology, surface roughness, and mechanical properties were studied by utilizing two chemical blowing agents (CBAs) with different activation temperatures and varying packing times. At the highest foaming temperatures, BMWD LCB PP foams exhibited 887% higher cell density, 46% smaller cell sizes, and more uniform cell structures than BWMD linear PP. Linear PP was found to have a surface roughness 23% higher on average than other resins. The BMWD LCB PP was found to have increased flexural modulus (44%) at the cost of decreased toughness (−88%) compared to linear PP. The branched architecture and high molecular weight of the BMWD LCB PP contributed to improved foam morphologies and surface quality in high-temperature MO-FIM conditions.

## 1. Introduction

Polymeric foams can have enhanced mechanical [1], thermal, and acoustic [2] properties, as well as significant reductions in weight in comparison to their solid counterparts, owing to the porous nature and cellular structure of the material. This combination of properties has seen cellular foams incorporated into several areas such as automotive parts, food packaging, and insulation. As such, researchers have sought to produce foams out of an increasing range of polymers.

Among commodity plastics, polypropylene (PP) is a low-cost thermoplastic that has a high service temperature and high chemical resistance [3]. It also has improved mechanical properties over other common thermoplastics such as polystyrene (PS) [4], and thus has been widely used in various foaming applications. However, typical PP has been found to produce low-quality foams with low cell density and large cells, which are attributed to its low melt strength and lack of strain hardening under extensional flow. These properties result in cell coalescence as cell nucleation and growth take place [5,6], particularly during foaming under high-temperature conditions.

Much work has been done to circumvent the shortcomings of PP and improve its foaming behavior. These efforts include inducing cross-linking, mixing PP with high-melt-strength (HMS) polymers, and changing the molecular structure of PP. Cross-linking has been shown to increase the foamability of the PP, and the degree of cross-linking was correlated to an increase in cell density and suppression of cell coalescence [7]. However, the formation of cross-links makes PP non-recyclable, which is a detriment to those seeking recyclable thermoplastics. Mixing linear PP with polymers such as high-density polyethylene (HDPE) is also a common solution that has been shown to increase the overall melt strength and improve cell morphology [8,9]. However, miscibility issues may be present when mixing different polymers, and controlling phase morphology becomes an additional concern and variable in foaming. Long-chain branching is another method to improve the foamability of PP. Long-chain branched (LCB) PP has been shown to have increased melt strength and exhibit strain hardening, [10,11] which result in smaller, denser cells, and a suppression of cell coalescence [10,12]. However, such advantages come at a cost—LCB PP is more expensive than linear PP.

A significant amount of research on improving PP foamability is focused on low melt flow rate (MFR) and low molecular weight distribution (MWD) polymers that are primarily used for extrusion foaming to manufacture foamed products with simple geometries, such as foam rods and sheets [13,14]. To create complex three-dimensional shapes with high expansion and good dimensional stability, mold-opening foam injection molding (MO-FIM) is used, which is a continuous foaming technique very similar to traditional injection molding but with the addition of a blowing agent and a foaming stage. MO-FIM consists of multiple steps: (1) gas is dissolved into molten polymer to create a homogenous solution, (2) this polymer/gas solution is injected into a mold, (3) a packing pressure is applied to suppress any premature cell nucleation, and (4) a pressure drop is induced by opening the mold quickly to a predefined distance. For easy processability in injection molding conditions, high-MFR polymers are used. However, high-MFR polymers typically have low melt strength and introduce additional challenges to foaming. Currently, there is a lack of commercially available high-MFR injection molding grade branched PP resins to fill this need for easy injection molding processing and improved foamability.

A major disadvantage of MO-FIM is the negative effects on surface quality, which appear as swirl marks, silver streaks, and surface blistering. These visual imperfections are a major hurdle for the use of foam-injection-molded products in applications where surface quality and surface appearance are critical factors. Silver streaks have been shown to be a result of cells prematurely nucleating during injection, which are then pushed towards the mold wall and elongated as the mold is filled [15]. As the melt rapidly cools upon coming in contact with the mold that is set to a relatively lower temperature, it solidifies and encapsulates these deformed cells on the surface, resulting in the appearance of long, thin, silver marks.

Previous work to reduce surface defects has focused on changes in processing conditions such as increased mold temperature [15] and decreased gas loading [16]. Unfortunately, these solutions both present challenges. To completely remove surface defects, it was found that the temperature of the mold needed to be at or above the crystallization temperature during injection [15], which would significantly affect cycles times. In addition, reduction in gas loading was shown to result in coarser cellular structures and reduce possible lightweighting. Some studies have demonstrated that an increase in melt strength through blending high-melt-strength polymers can improve the surface quality of FIM parts due to a finer cellular structure [17], as it is understood that cells nucleated upon injection are so small that the visual effects on the surface are much less pronounced even when the bubbles become elongated. However, no known studies to date have focused on comparisons between LCB PP and linear PP on the surface quality of MO-FIM parts.

Motivated by the lack of research into the effect of molecular architecture on surface quality in MO-FIM involving high-MFR polymers, this work evaluates the use of a broad molecular weight distribution (BMWD) linear PP and BMWD LCB PP resin in a MO-FIM process with chemical blowing agents (CBAs). These resins are compared to a commercial linear PP with low MWD to determine how changes in molecular structure and MWD affect cellular morphology, surface quality, and mechanical properties. Further, a systematic study of various processing conditions is conducted to determine their influence on foam properties, and whether the degree of influence varies among polymer resins. Two CBAs with different activation temperatures are employed to further analyze how foam properties are influenced by processing temperatures.

It was found that the BMWD-LCB-PP-produced foams with higher cell densities and smaller cells than the BMWD linear PP and the commercial linear PP under high-temperature foaming conditions. The improvements seen in BMWD LCB PP are attributed to increased extensional viscosity as a result of strain hardening caused by the branched structure of the polymer. The BMWDLCB PP also produced notably “smoother” cells than the other tested resins. The BMWD resins were both found to have smoother surfaces than the linear PP. The BMWD LCB PP was also found to have increased flexural strength and modulus relative to the linear PP. However, it was found to be much more brittle with a significant reduction in toughness.

## 2. Materials and Methods

### 2.1. Materials and Sample Preparations

This study utilized three PP resins provided by ExxonMobil Chemical Company (Houston, TX, USA): a commercial linear PP, a BMWD linear PP grade, and a BMWD LCB PP grade. The molecular properties of these resins are noted in Table 1.

The resins were used as received in the foaming experiments. For foaming, two different CBAs were utilized: a high-activation-temperature CBA and low-activation-temperature CBA provided by Uniform Color Company (Holland, MI, USA) and Avient Corporation (Avon Lake, OH, USA), respectively. Properties for these CBAs are noted in Table 2.

To test different CBA loading conditions, batches of PP and CBA were dry mixed before use in MO-FIM. Batches were created for each CBA–resin combination at 1 wt% and 2 wt% CBA, resulting in a total of 12 different formulations. The batches of material are denoted as “resin type-CBA wt%-CBA type”. For example, A-1-AV represents the batch of Resin A with 1 wt% AV CBA.

### 2.2. Foam Injection Molding and Processing Conditions

**Figure 1:**
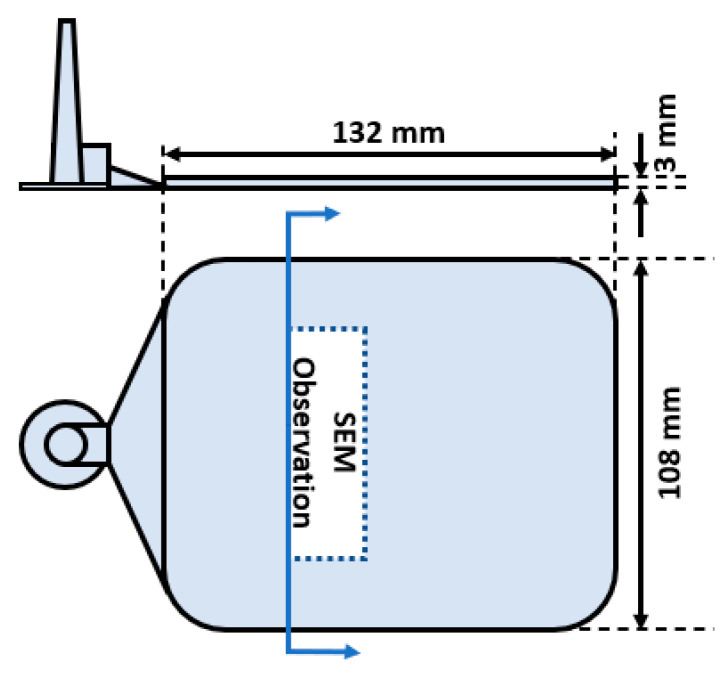
Schematic of the mold dimensions with the general area of SEM observations labelled.

A 50-ton injection molding machine (Arburg Allrounder 270, ARBURG Inc., Loßburg, Germany) and a two-plate mold were used to conduct MO-FIM experiments. Figure 1 illustrates a schematic of the mold used, along with the area in which scanning electron microscopy (SEM) characterization was conducted and surface measurements were taken.

Detailed information on processing conditions is listed in Table 3. The majority of parameters were fixed, while the packing time was varied from 0.5 s to 20 s. A range of packing times was explored as a means to effectively vary the foaming temperature, which is the melt temperature when foaming is induced (i.e., when the mold opens in MO-FIM). Such test conditions were designed to study the effect of melt temperature on foam properties, as the melt is expected to remain molten at shorter packing times and be cooled further at longer packing times. An elevated mold temperature of 80 °C was used to improve surface quality of the foamed product and to reduce the thermal gradient from the nozzle to the mold, thereby slowing down the cooling of the melt to ultimately pronounce the effect that packing time had on foaming temperature. A mold-opening distance of 0.5 mm was used to achieve an expansion ratio of ~1.17, or in other words a void fraction about 14%.

Due to difference in activation temperature for both CBAs, different barrel temperature profiles were required. The barrel was divided into six different heating zones labeled as Zone 1 through Zone 6, with Zone 1 being closest to the hopper and Zone 6 being closest to the nozzle (and the mold). The different temperature profiles are summarized in Table 4.

### 2.3. Differential Scanning Calorimetry

A differential scanning calorimeter (DSC) (DSC 250, TA instruments INC., New Castle, DE, USA) was used to investigate the melting and crystallization temperatures of all three resins. A testing sample of about 10 mg was placed between an aluminum pan and cap, within a nitrogen-filled chamber. The samples were first heated to 50 °C, then to 250 °C at a rate of 10 °C/min and isothermally kept for 10 min for the elimination of thermal history. The sample was then cooled to 50 °C at a rate of 10 °C/min and heated again to 250 °C.

### 2.4. Rheological Characterization

Rheological characterization was performed to reveal the degree of shear thinning or strain hardening in the resins tested, as the viscoelastic properties of the material have a significant influence on the foaming process by impacting the energy barrier in cell nucleation and controlling cell coalescence or collapse in cell growth. A rotational rheometer (ARES-G2, TA Instruments Inc., New Castle, DE, USA) with a 25 mm parallel plate geometry was used to conduct small amplitude oscillatory shear measurements at 190 °C. Strain sweep experiments were first performed to determine the linear viscoelastic region, and frequency sweep measurements were carried out from 0.01 to 100 rad/s at 10% applied strain.

The same rheometer was used to conduct uniaxial elongation viscosity measurements by equipping an extensional viscosity fixture (SER3-A, Xpansion Instruments, Spicewood, TX, USA). Measurements were carried out at 175 °C with three levels of extension rates (0.1, 1.0, and 10 s^−1^) up to a Hencky strain of 3.

### 2.5. Surface Roughness Characterization

Surface roughness was measured using a hand-held surface roughness tester (TR200, Beijing TIME High Technology Ltd., Beijing, China). Measurements were obtained within an area similar to the SEM measurement zone shown in Figure 1, but on the surface of the parts. Measurements were taken on 5 samples, and the mean Rz value is reported. Rz is the average value of several maximum peak-to-valley height measurements of a surface over successive evaluation lengths [18].

### 2.6. Cell Morphology Characterization

To characterize the morphology of the cellular structures within the foam, a scanning electron microscope (SEM) (Phenom Pro, Thermo Fisher Scientific, Waltham, MA, USA) was used. To prepare the SEM specimens, part of the foamed sample was cryogenically fractured after a 15-min immersion in liquid nitrogen. The fractured specimen was then coated with a roughly 10 nm layer of platinum by a sputter coater (SC7620, Quorom Technologies Ltd., Laughton, UK) to prevent charging of the sample surface. Based on the obtained SEM images, the cell count and cell size were characterized using ImageJ software. The cell sizes are reported as average cell size of at least 30 cells. The cell density (N) is calculated according to the following equation.

N = (n/A)^3/2^ Ψ (1)

where n is the counted number of cells within a specific area A, and Ψ is the expansion ratio, which is the ratio of the volume of a foamed sample over the volume of a solid sample. Since the mold only expands in one direction, Ψ was obtained by measuring the total thickness of the foamed portion of the sample and dividing by the original depth of the mold (3 mm) minus the skin thickness.

The thickness of the skin layer, defined as the solid section containing no cells, was determined by analyzing the same cross sections used for cell morphology characterization. The skin thickness was measured as the shortest distance from the surface to a cell. For each test condition, the reported skin thickness is the mean value of twelve measurements—six measurements each from two specimens.

### 2.7. Mechanical Property Evaluation

Flexure properties were evaluated using a three-point bending fixture on a tensile testing machine (Instron 5965, Instron, Norwood, MA, USA). Flexural tests were conducted by cutting flexural test specimens out of injected molded samples aligned parallel to the direction of melt flow. Values for span and displacement rate were calculated based on measured specimen dimensions and equations found in ASTM D790-17. The mean values of three specimens from two different injection molding samples (for a total of six specimens) are reported for ultimate flexural strength, flexural modulus, and toughness. The reported flexural modulus is the 1% strain secant modulus.

Testing was conducted beyond the 5% strain limit described in ASTM D790-17 to measure the toughness of all samples, since Resin A did not achieve complete failure in almost all tested conditions. If material failure was not achieved beyond 5% strain, the test was concluded after reaching a 20% drop in force from the maximum, which was designated as the failure point.

## 3. Results and Discussion

### 3.1. Crystallization and Melting Temperatures

**Figure 2:**
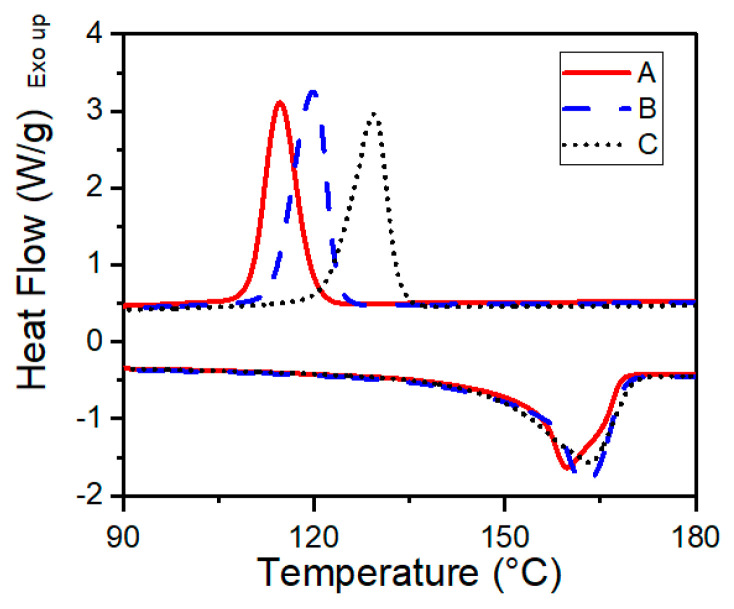
DSC curves for all three resins. Heating and cooling conducted at 10 °C/min.

The peak melting temperature of all three resins was found to be similar, as shown in Figure 2. Additional data gathered from DSC testing is summarized in Table 5. Resins A, B, and C were found to have peak melting temperatures of 159.7 °C, 162.6 °C, and 163.3 °C, respectively. Significant differences were found in crystallization temperatures (*T_c_*): peak crystallization temperatures of Resin A (114.7 °C) and Resin B (119.8 °C) were significantly lower than that of Resin C (129.3 °C). This higher crystallization temperature of Resin C is a result of its branched structure that serves as heterogeneous crystal nucleation sites [13,19,20].

### 3.2. Shear and Extensional Rheology

**Figure 3:**
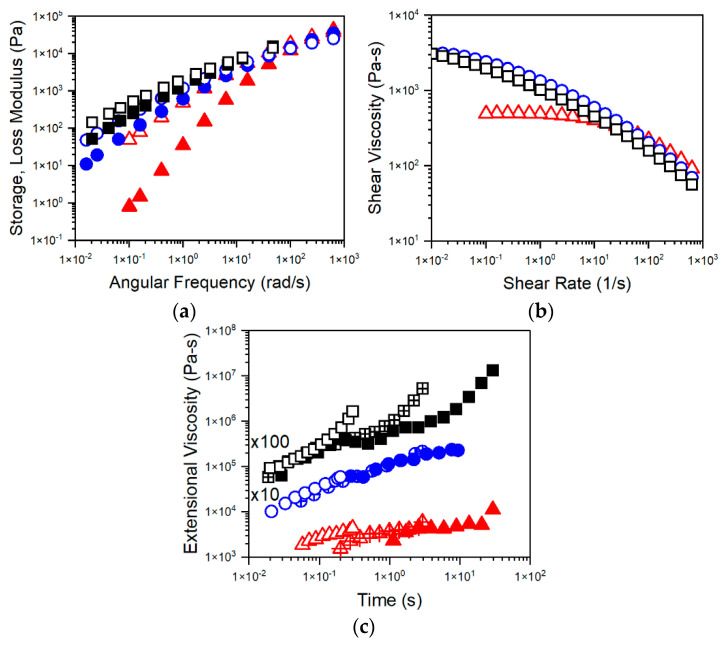
Rheology of Resin A (red triangles), Resin B (blue circles), and Resin C (black squares). (**a**) Storage (solid) and loss (hollow) moduli at 190 °C. (**b**) Estimated shear viscosity via Cox–Merz rule at 190 °C. (**c**) Extensional viscosity of Resin A, B (×10), and C (×100) at 175 °C using strain rates of 0.1 s^−1^ (solid), 1 s^−1^ (internal cross), and 10 s^−1^ (hollow).

Frequency sweep results showed that all three resins have comparable storage and loss moduli at high frequencies (Figure 3a). However, Resin A exhibited the fastest relaxation and reached the terminal region, as reflected in the quick transition from an elastic response at high angular frequencies to a predominantly viscous response at low frequencies. In contrast, Resins B and C did not seem to have fully relaxed over the range of frequencies explored. These observations are consistent with the fact that Resin A has a narrow molecular weight distribution, while Resins B and C both have a wide molecular weight distribution (Table 1). Such difference in polydispersity was also reflected in the shear viscosity measurements estimated from the complex viscosity using the Cox–Merz rule (Figure 3b). While similar degrees of shear thinning were observed in all three resins at high shear rates, which are relevant to processing conditions in FIM, the zero-shear viscosity of Resin A was lower than those of Resins B and C by nearly an order of magnitude. This difference is attributed to the lower molecular weight of Resin A compared to those of the other two resins. In fact, the similarity between molecular weight and molecular weight distribution of Resins B and C led to such minimal difference in their viscoelastic response under shear. However, a distinct difference between Resins B and C was seen in extensional rheology, where only Resin C exhibited strain hardening owing to its branched structure (Figure 3c).

### 3.3. Cell Morphology

**Figure 4:**
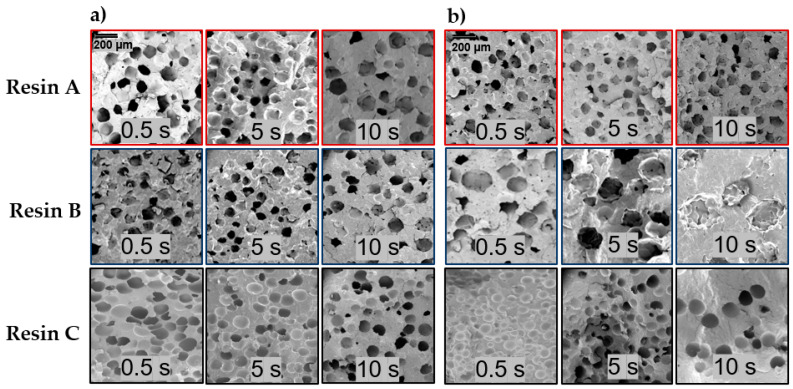
SEM image comparison of cellular morphology for Resins A, B, and C when foamed with (**a**) 2 wt% AV CBA and (**b**) 2 wt% UC CBA at different packing times (0.5, 5, and 10 s).

Based on SEM images in Figure 4, the shape and texture of cells are different amongst the resins. Resin C demonstrates smooth and uniform cell surfaces, in contrast to Resins A and B which exhibit irregular cell shapes and surfaces, particularly Resin B. The differences in cells become more pronounced towards longer packing times. The different cellular structure is attributed to the branched structure in Resin C which results in a higher T_c_ and faster crystallization rate [21]. This crystallization behavior leads to earlier solidification of the melt and in combination with strain hardening improves cell stabilization during cell growth. Additionally, branched PP resins have been found to have decreased spherulite sizes [22], and differences in spherulite size and structure have been shown to affect cell morphology of foamed PP [23]. Specifically, the cell wall morphologies seen in the SEM images of Resins A and B (Figure 4) are a result of cells expanding past growing spherulites during cell growth [24]. The amorphous melt between these spherulites is pushed away by the expanding gas, resulting in the uneven texture. The cell walls in Resin C appear to be much smoother because of simultaneous cell growth and cell wall stabilization (via crystallization of the melt), owing to the faster crystallization rate. Therefore, there is little or no melt between spherulites to push away, resulting in no noticeable texture at the visual scales shown.

**Figure 5:**
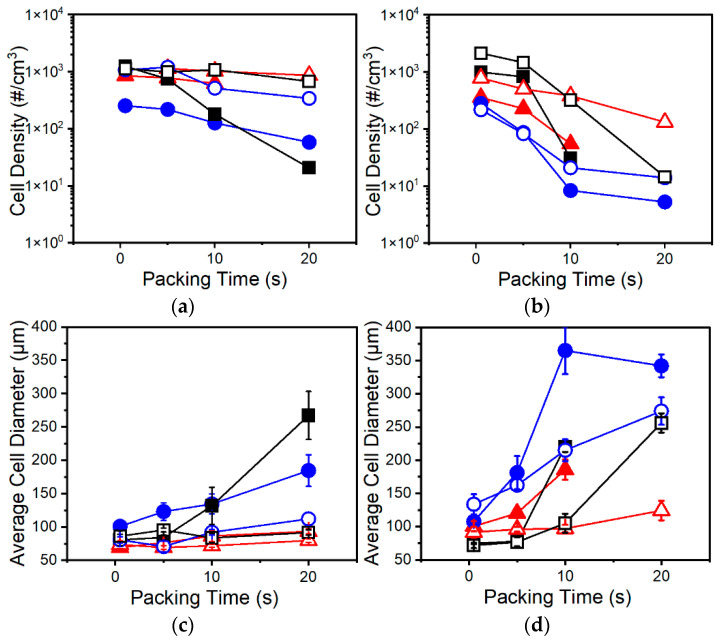
Cell density (**a**,**b**), and average cell diameters (**c**,**d**) of Resin A (red triangles), Resin B (blue circles), and Resin C (black squares) samples foamed using: (**a**,**c**) AV CBA and (**b**,**d**) UC CBA under various packing times. Closed and open symbols indicate 1 wt% and 2 wt% CBA, respectively.

**Figure 6:**
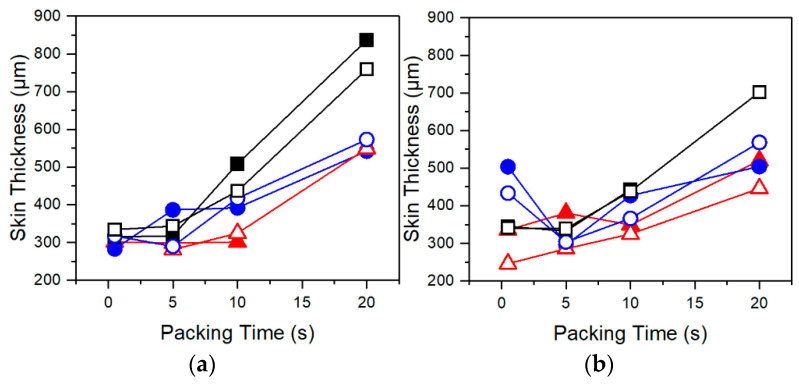
Skin thickness of Resin A (red triangles), Resin B (blue circles), and Resin C (black squares) samples foamed using (**a**) AV CBA and (**b**) UC CBA under various packing times. Closed and open symbols indicate 1 wt% and 2 wt% CBA, respectively.

Inspecting the results of cell morphology shown in Figure 5 demonstrates that amongst all resins cell density decreases, and cell sizes increase with longer packing times. At increased packing times, the melt is much cooler, and a larger portion of the melt has solidified, as evidenced by increased skin thickness with longer packing times in Figure 6. These trends suggest that crystallization is playing a dominant role in controlling the foaming process. An excessively cool melt has been shown to hinder cell nucleation and growth through decreased gas diffusivity and increased melt stiffness [3]. Evidence of suppressed cell nucleation is reflected in the decrease in cell density with increasing packing time in all resins (Figure 5a,b). Further, the higher cell density, smaller cell sizes, and decreased sensitivity to packing time at higher CBA wt% can be partly attributed to decreased T_c_ of PP due to CO_2_ plasticization [13,14,25], which is further evidence that foaming under these test conditions is dominated by crystallization. From these trends, it can be postulated that the injected melt subjected to a longer packing time has been excessively cooled to a point that a larger portion of the polymer has solidified and lost more of its capability to foam upon mold opening. In other words, a decrease in cell density with increasing packing time may be the result of a larger amount of CO_2_ being expelled from the crystal lattice as crystallization continues.

Lower foaming temperatures also contribute to larger average cell sizes seen at longer packing times (Figure 5c,d) through several means. First, the melt temperature is reduced at longer packing times such that cell nucleation is suppressed during foaming. Therefore, during foaming, CO_2_ will preferentially diffuse to existing cells nucleated early during mold opening rather than newly nucleated cells. This effect is compounded by a growing skin which expels dissolved CO_2_ as crystallization occurs, providing further amounts of gas for cell growth in the adjacent core. Second, increased cell sizes may also be a result of the increased effective expansion ratio. At higher packing times, a thicker skin has formed (Figure 6) and the core is a smaller portion of the overall mold thickness, but the set mold expansion is the same. Thus, upon mold opening a thinner core must now expand further to compensate for the non-expanding skin to achieve the set expansion. This effectively increases the expansion ratio for the molten core. Excessive expansion then results in increased possibility of cell coalescence due to increased extensional stresses/strains. In summary, increased packing times contribute to increased cell diameters by lowering melt temperatures such that gas preferentially contributes to cell growth rather than cell nucleation, and by increasing the effective expansion ratio of the core thereby inducing cell coalescence through increased extensional stresses/strains.

Distinct differences in foaming behaviors can be seen amongst the resins. Resin C demonstrates improved foaming relative to Resins A and B when foamed with UC CBA at low packing times—as shown in the lower cell density and larger cells of Resins A and B (Figure 5b,d). This relative improvement is attributed to a deterioration in foaming in Resins A and B at the higher foaming temperatures present when using UC CBA. UC CBA has higher foaming temperatures compared to AV CBA because of higher peak barrel temperatures, resulting in higher melt injection temperatures despite the set injection temperatures being the same for both CBAs. The increased injection temperature of UC CBA results in higher foaming temperatures for the same packing times compared to AV CBA, which lowers melt strength and increases the degree of cell coalescence in Resins A and B. In contrast to Resins A and B, Resin C sees better foaming at higher foaming temperatures due to higher melt strength and the presence of strain hardening due to branching. Both properties act to suppress coalescence and improve cell stability, resulting in the higher cell densities for C-1-UC and C-2-UC below packing times of 10 s. Improvements in foaming due to the branched structure of Resin C are in accordance with existing literature on low-MFR LCB PP exhibiting better foaming behavior relative to linear PP with similar MFR [6,12].

Additionally, differences in foaming behaviors amongst resins between UC and AV CBA can be attributed to increased gas yield in AV CBA. As noted earlier, higher amounts of dissolved gas allow for increased cell density and smaller cell sizes [14]. The higher amounts of dissolved CO_2_ also lower T_c_ of PP [13,25] which decreases sensitivity to packing time amongst the resins. This is especially prominent in Resin C, where the increased T_c_ due to branching results in drastic decreases in cell densities and much larger cells at packing times higher than 5 s. At packing times above 5 s, Resin A exhibited the highest cell densities and smallest cell sizes for comparable CBA loading. The exception to these trends is the C-2-AV batch (Figure 5a,c). This batch theoretically has the highest degree of dissolved CO_2_ and therefore the lowest T_c_ among Resin C batches, which explains the decrease in sensitivity to foaming temperature as shown by a lack of distinct drop in cell density or increase in cell size at longer packing times.

Resin B stands out as the PP resin with the lowest cell densities and larger cell sizes at most tested conditions. With a higher melt strength than Resin A due to high M_w_ and broad MWD comparable to those of Resin C, it was expected that Resin B would have improved foaming characteristics. It is believed that the poor foaming behavior stems from a lower CO_2_ solubility in Resin B due to its broader molecular weight distribution [26]. The low solubility, in combination with an already relatively low amount of CO_2_, resulted in the significant differences between Resins A and B as less gas is dissolved and available for foaming.

### 3.4. Surface Roughness

**Figure 7:**
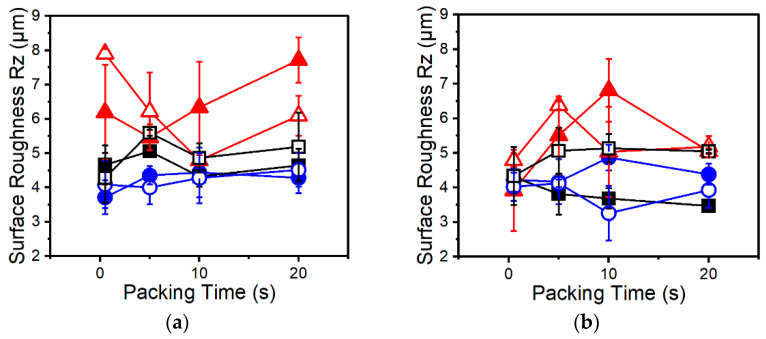
Surface roughness of Resin A (red triangles), Resin B (blue circles), and Resin C (black squares) samples foamed using (**a**) AV CBA and (**b**) UC CBA under various packing times. Closed and open symbols indicate 1 wt% and 2 wt% CBA, respectively.

No clear trends were found with regards to surface roughness and packing time, as shown in Figure 7. This is because the outer layer of the skin is formed almost immediately upon injection as the melt touches the relatively cooler mold walls. However, general conclusions can be drawn amongst the PP resins. Resin A has mostly higher surface roughness than Resins B and C. It also has a higher degree of variability in measurements as evidenced by the larger error bars. This is attributed to the low melt strength and particular foaming behavior of Resin A. Due to a high cell density (Figure 5a,b), it is inferred that Resin A experiences a higher degree of premature cell nucleation during injection. These prematurely nucleated cells are expected to become more elongated and deformed as the cells are pushed towards the surface of the mold than cells in a BMWD PP such as Resins B or C, due to the low melt strength of Resin A. Thus, a high degree of premature cell nucleation and low melt strength would cause larger, more numerous imperfections on the surface of the melt. As a result, a rougher surface is measured with a higher degree of variability. However, this trend is not reflected in cell density measurements (Figure 5a,b) since the application of packing pressure shrinks and redissolves the premature cells within the core while the outer skin layer remains unaffected and maintains the imperfections. Further, if Resin B does in fact have lower solubility as discussed earlier, then the improved surface roughness of Resin B may be in part due to fewer cells nucleated during injection and not purely due to improved melt strength.

Due to elevated mold temperatures the surface of the foamed parts is already improved to some degree relative to an unheated mold. This improvement diminishes the differences amongst the resins with regards to effects on surface roughness. To better capture differences amongst resins on surface roughness, it may be best to examine the effects of these resins at various mold temperatures rather than one condition as done so here.

### 3.5. Flexural Properties

**Figure 8:**
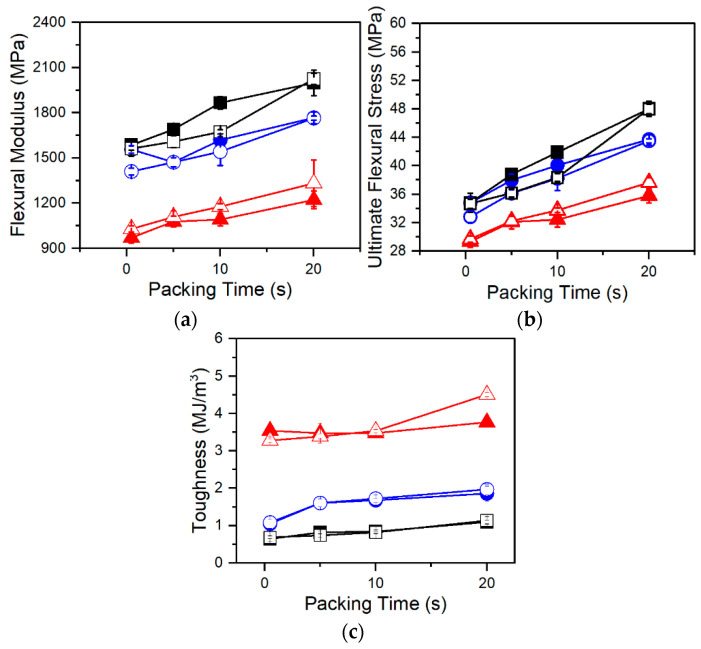
(**a**) Flexural modulus, (**b**) ultimate flexural strength, and (**c**) toughness of Resin A (red triangles), Resin B (blue circles), and Resin C (black squares) foamed at various packing times using AV CBA. Closed and open symbols indicate 1 wt% and 2 wt% CBA respectively.

**Figure 9:**
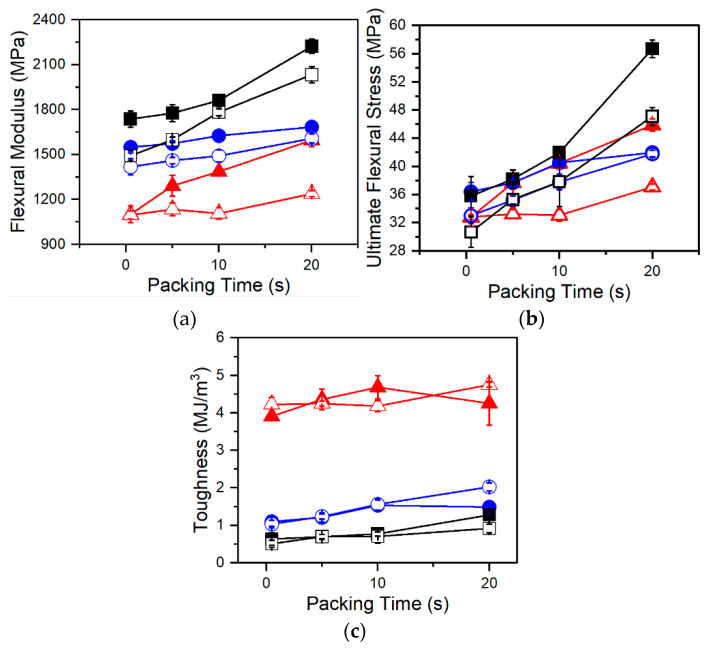
(**a**) Flexural modulus, (**b**) ultimate flexural strength, and (**c**) toughness of Resin A (red triangles), Resin B (blue circles), and Resin C (black squares) foamed at various packing times using UC CBA. Closed and open symbols indicate 1 wt% and 2 wt% CBA respectively.

Flexural testing of foamed samples demonstrated consistent trends amongst each resin. In Figure 8 and Figure 9, flexural modulus and ultimate flexural strength are shown to increase with increasing packing times. This is a result of changing foam structures. The MO-FIM samples consist of distinct layers: the solid outer skin and the foamed inner core. The flexural modulus of the MO-FIM sample is dependent on the combined rigidity of the foamed core and the solid skin with their contributions to the flexural modulus, dependent on the ratio of their depth to the overall thickness. This is described by the rigidity mixing rule equation for sandwich foams [27]:

F = (δ_c_/δ)^3^(1 − f_c_)^2^E_m_ + (1 − (δ_c_/δ)^3^)E_m_, (2)

where F is the flexural modulus of the injection molded sample, E_m_ is the flexural modulus of the solid polymer, δ_c_ is the thickness of the foamed core, δ is the total thickness of the sample, and f_c_ is the void fraction of the foamed core. With increased packing time there is an increase in skin thickness (Figure 6) which decreases the ratio of foamed core to total thickness. According to Equation (2), this would increase the stiffness contribution of the skin and ultimately increase the flexural modulus of the samples. This trend is consistent with the data shown in Figure 8a and Figure 9a. Differences in foam structures amongst resins also influence the sensitivity of the flexural modulus and strength to packing time. For example, in Figure 9a,b, the flexural modulus and strength of Resin C increases at a much faster rate at later packing times than those of either Resin A or B. This increased sensitivity is because the foam morphology and skin thickness of Resin C are highly sensitive to long packing times due to increased T_c_. The result is a thicker skin and denser core at longer packing times, leading to the increases in flexural modulus and strength.

However, differences amongst the resins independent of foaming conditions are clear. The cell morphologies of A-2-AV, B-2-AV, and C-2-AV were very similar in terms of cell densities and cell size (Figure 5a,c), but when comparing their flexural properties (Figure 8a,b), the resins show distinct differences in flexural modulus and strength. Resins B and C are stiffer, stronger materials, as shown by the higher flexural modulus and ultimate flexural strength, with Resin C being slightly stiffer and stronger than Resin B. The differences amongst the resins are best presented when analyzing toughness. Toughness is seen to have a decreased sensitivity to packing time and therefore changing foam morphology, making toughness largely dependent on material properties. Resin A is seen to have much higher toughness (and therefore ductility) than either Resin B or C, and Resin B is slightly more ductile than Resin C.

Branched PP has been shown to have smaller spherulites than linear PP in the presence of dissolved CO_2_ and shear flow [13]. Smaller spherulites and molecular entanglements contribute to increased tensile modulus in branched polymers over their linear counterparts [21]. But despite being linear in structure, Resin B demonstrated similar flexural properties to Resin C. Since Resins B and C share similar molecular weight attributes, these differences in mechanical properties between Resin A and Resins B and C are then largely attributed to the increased molecular weight and broader molecular weight distribution. The role of branching is smaller, though not insignificant, since Resin C is shown to have increased strength and rigidity along with decreased ductility over Resin B.

## 4. Conclusions

In this work, three different PP resins were investigated to determine foaming characteristics, mechanical properties, and effects on surface quality in MO-FIM. A linear (Resin A), a BMWD linear (Resin B), and a BMWD LCB PP (Resin C) were foamed under various packing times and with two CBAs with different activation temperatures.

It was determined that the branched structure of Resin C increased cell densities and decreased cell sizes relative to Resins A and B at high foaming temperatures. At 2 wt% UC CBA loading with 0.5 s of packing time, Resin C has 173% and 887% higher cell density, and 22% and 46% smaller cell sizes than Resins A and B, respectively. However, the high T_c_ resulted in sensitivity to low foaming temperature and low gas loading, whereas at these conditions Resin A was found to produce the highest cell densities (620 cells/cm^3^ vs. 180 cells/cm^3^ for Resin C at 1 wt% AV, 10 s packing time). Resin B was expected to produce high quality foams due to broader molecular weight distribution than Resin A but instead produced the lowest cell densities and largest cells of all resins at most conditions. This is attributed to a decrease in real solubility due to a broadened molecular weight distribution while lacking strain hardening. Further, Resin A was found to have higher surface roughness and increased variability in measurements compared to Resins B and C. On average, Resin A had a 27% higher surface roughness than Resin B and a 19% higher surface roughness than Resin C. Additionally, it was found that although foam morphology played a role in flexural properties, molecular structure was the dominating factor in our processing conditions. The increased molecular weight and broader distribution of Resins B and C resulted in significant increases in flexural strength and rigidity at the cost of decreased ductility. The branched structure of Resin C further increased strength and rigidity, with decreased ductility. For example, at 2 wt% AV CBA loading and 0.5 s of packing time, Resin C foams demonstrated a flexural strength 17% and 6% higher, flexural modulus 52% higher and 11% higher, and toughness 79% and 37% lower than Resins A and B, respectively.

In conclusion, Resin C was found to have numerous benefits with improved high-temperature foaming, improved surface quality, and increased flexural strength even over Resin B which has similar molecular weight properties. High-temperature foaming performance can benefit large scale industrial MO-FIM due to increased shear heating, and improvements to surface quality are also promising for broadening applications for MO-FIM PP. However, drawbacks such as brittleness, high crystallization temperature, and high cost need to be addressed and mitigated to encourage wider use of high-MFR branched PP in FIM applications.

## Figures and Tables

**Table 1:** Molecular properties of three studied resins. M_w_ is the weight-average molecular weight, M_w_/M_n_ the molecular polydispersity index, and MFR is the melt flow rate. (Data from ExxonMobil Chemical Company).

Designation	Material	M_w_^1^ (g/mol)	M_w_/M_n_^1^ (g/mol)	MFR ^2^ (g/10 min)
A	Linear	182,000	3.7	37
B	BMWD Linear	303,000	18	30
C	BMWD Branched	292,000	19	40

^1^ Based on ExxonMobil test method EM 318 rev 1. ^2^ Based on test method ASTM D1238.

**Table 2:** Properties of the CBAs used in this study. Decomposition temperatures and %weight loss are determined from differential scanning calorimetry (Figure S1) and thermogravimetric analysis data (Figure S2), respectively.

Designation	Material	Gas	Decomposition Ranges (°C)		Weight Loss (%) ^1^
AV	ITP-817A	CO_2_	161–175	211–229	13.5
UC	ACBA35-450EN	CO_2_	157–175	193–215	9.9

^1^ Measured at high-temperature end of decomposition ranges.

**Table 3:** MO-FIM molding processing conditions.

Parameters	Value
Injection Speed (cm^3^/s)	100
Mold Temperature (°C)	80
Shot Size (cm^3^)	60
Screw Speed (rpm)	500
Barrel Pressure (MPa)	15
Packing Pressure (MPa)	20
Packing Time (s)	0.5, 5, 10, 20
Mold Opening Distance (mm)	0.5
Mold Opening Speed (mm/s)	50
Cooling Time (s)	60 s

**Table 4:** Barrel temperature profiles used for each CBA to ensure activation. All temperatures are in Celsius.

CBA	Zone 1 (Hopper)	Zone 2	Zone 3	Zone 4	Zone 5	Zone 6 (Nozzle)
UC	50	200	230	210	190	190
AV	50	200	215	200	190	190

**Table 5:** Summarized DSC results for all three resins: peak crystallization temperature (T_c_), peak melting temperature (*T_m_*), heat of fusion (ΔH_m_), and percent crystallinity (Χ_c_). Heating and cooling conducted at 10 °C/min. Crystallinity is calculated based on ΔH_m_ of 207 J/g for 100% crystalline PP.

Resin	*T_c_* (°C)	*T_m_* (°C)	Δ*H_m_* (J/g)	*Χ_c_* (%*)*
A	115	160	103	49
B	120	163	111	54
C	129	163	110	53

## Supplementary Materials

The following are available online at https://www.mdpi.com/article/10.3390/polym13152404/s1, Figure S1: DSC curves for AV and UC CBA as received, Figure S2: TGA curves for AC and UC CBA indicating weight % from 50 °C to 250 °C.
