# Supplementary material for: Improved Cell Morphology and Surface Roughness in High-Temperature Foam Injection Molding Using a Long-Chain Branched Polypropylene
Source: Polymers (Basel). 2021 Jul 22;13(15):2404. doi: 10.3390/polym13152404 (PMC8348131; doi:10.3390/polym13152404)
Supplement: Supplementary file 1 [file polymers-13-02404-s001.zip › polymers-1293924-supplementary.pdf]

polymers MDPI

Article

Improved Cell Morphology and Surface Roughness in High-Temperature Foam Injection Molding Using a Long-Chain Branched Polypropylene

Steven Mendoza-Cedeno [1], Mu-Sung Kweon [1], Sarah Newby [2], Maksim Shivokhin [2], George Pehlert [2] and Patrick C. Lee [1,*]

[1] Department of Mechanical and Industrial Engineering, University of Toronto, 5 King's College Road, Toronto, ON M5S 3G8, Canada; smendoza@mie.utoronto.ca (S.M.-C.); mkweon@mie.utoronto.ca (M.S.K.)
[2] ExxonMobil Chemical Company, 5200 Bayway Drive, Baytown, TX 77520, USA; sarah.newby@exxonmobil.com (S.N.); maksim.e.shivokhin@exxonmobil.com (M.S.); george.j.pehlert@exxonmobil.com (G.P.)
* Correspondence: patricklee@mie.utoronto.ca; Tel.: +1-(416)-946-5407

Citation: Mendoza-Cedeno, S.; Kweon, M.S.; Newby, S.; Shivokhin, M.; Pehlert, G. Improved Cell Morphology and Surface Roughness in High-Temperature Foam Injection Molding Using a Long-Chain Branched Polypropylene. *Polymers* **2021**, *13*, 2404. https://doi.org/10.3390/polym13152404

Academic Editor: Luigi Sorrentino

Received: 25 June 2021
Accepted: 17 July 2021
Published: 22 July 2021

Publisher's Note: MDPI stays neutral with regard to jurisdictional claims in published maps and institutional affiliations.

Copyright: © 2021 by the authors. Licensee MDPI, Basel, Switzerland. This article is an open access article distributed under the terms and conditions of the Creative Commons Attribution (CC BY) license (http://creativecommons.org/licenses/by/4.0/).

Differential Scanning Calorimetry (DSC)

DSC analysis was conducted determine the range of activation temperatures for both chemical blowing agents (CBAs). The samples were heated to 50 °C, isothermally kept for 10 min, then heated to 300 °C at a rate of 10 °C/min.

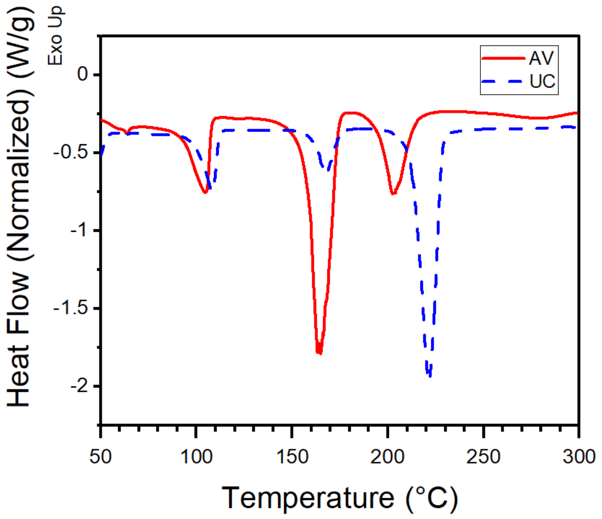

Figure S1. DSC curves for AV and UC CBA as received at a heating rate of 10 °C/min.

The first melting curve for both resins at around 100 °C is indicative of the carrier resin melting. The later curves indicate the active components in the CBAs decomposing and producing gas. The first endothermic peak corresponds to sodium bicarbonate decomposition. The second endothermic peaks correspond to decomposition of citric acid. Based on these results, both CBAs appear to contain a combination of sodium bicarbonate and citric acid as active ingredients, although in different quantities. AV CBA appears to have a substantial sodium bicarbonate component, whereas UC has a substantial citric acid component. Based on these results, the peak barrel temperatures for AV and UC CBA were chosen as 215 °C and 230 °C, respectively.

Thermogravimetric Analysis (TGA)

A TGA instrument (TGA Q50, TA Instruments Inc., New Castle, DE, USA) was used to determine the weight percentage of gas released by either CBA. For testing, the materials were heated to 50 °C, then heated to 250 °C or 300 °C for AV and UC CBA respectively, at a rate of 10 °C/min.

The results from Figure S2 show that at the peak barrel temperatures, AV CBA releases a higher weight percentage of gas than UC CBA. AV CBA has a weight loss of 13.5% and UC CBA has a weight loss of 9.9%. The reactions for citric acid and sodium bicarbonate both release a 1:1 mol ratio of CO_2 and H_2O in their decomposition reactions [1], therefore the proportion of weight loss made up of CO_2 is the same for UC and AV CBA. This demonstrates that AV CBA is releasing a larger amount of CO_2 into the polymer melt than UC CBA at the respective peak barrel temperatures used in this study.

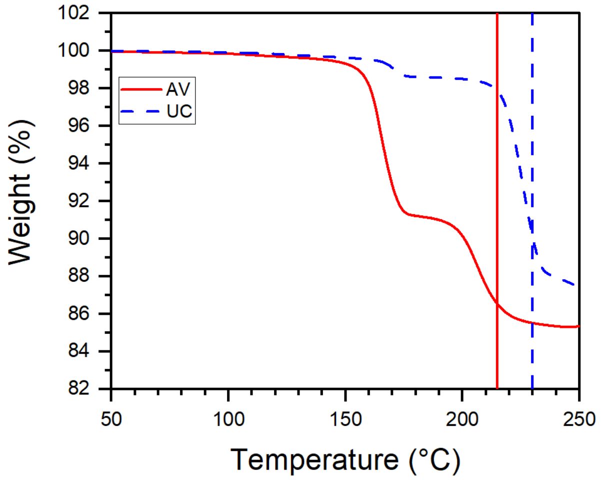

Figure S2. TGA curves for AV and UC CBA indicating weight % from 50 °C to 250 °C at a heating rate of 10 °C/min. Vertical lines indicate peak barrel temperatures for the respective CBA.

Reference

1. Ruiz, J.A.R.; Vincent, M.; Agassant, J.-F.; Sadik, T.; Pillon, C.; Carrot, C.; Polymer foaming with chemical blowing agents: Experiment and modeling. *Poly. Eng. Sci.* **2014**, *55*, 2018–2029, doi:10.1002/pen.24044.
